# Impact of a Low-Insulin-Stimulating Bread on Weight Development—A Real Life Randomised Controlled Trial

**DOI:** 10.3390/nu15051301

**Published:** 2023-03-06

**Authors:** Kerstin Kempf, Martin Röhling, Hubert Kolb, Stephan Martin

**Affiliations:** 1 West-German Centre of Diabetes and Health, Düsseldorf Catholic Hospital Group, 40591 Düsseldorf, Germany; 2 Faculty of Medicine, Heinrich-Heine-University Düsseldorf, 40591 Düsseldorf, Germany

**Keywords:** bread, carbohydrate, glucose, insulin, overweight, weight reduction

## Abstract

The impact on body weight development is usually analysed by comparing different diet types. Our approach was to change only one component, namely bread, common to most diets. In a single-centre triple-blind randomised controlled trial the effects of two different breads on body weight were analyzed without further lifestyle modification. Overweight adult volunteers (*n* = 80) were randomised 1:1 to exchange previously consumed breads for either a rye bread from milled whole grain (control) or a medium-carbohydrate, low-insulin-stimulating bread (intervention). Pre-tests demonstrated that the two bread types strongly differed in the glucose and insulin response elicited, but had similar energy content, texture and taste. The primary endpoint was the estimated treatment difference (ETD) in change of body weight after 3 months of treatment. Whereas body weight remained unchanged in the control group (−0.1 ± 2.0 kg), significant weight reduction was observed in the intervention group (−1.8 ± 2.9 kg), with an ETD of −1.7 ± 0.2 kg (*p* = 0.007), that was more pronounced in participants ≥ 55 years (−2.6 ± 3.3 kg), paralleled by significant reductions in body mass index and hip circumference. Moreover, in the intervention group, the percentage of participants with significant weight loss (≥1 kg) was twice as high as in the control group (*p* < 0.001). No other statistically significant changes in clinical or lifestyle parameters were noted. Simply exchanging a common insulinogenic bread for a low-insulin-stimulating bread demonstrates potential to induce weight loss in overweight persons, especially those at older age.

## 1. Introduction

Bread is the most widely consumed grain-based food in the world and is also one of the largest sources of carbohydrate in the Western diet. In Europe, it provides up to 30% of the daily carbohydrate consumption in women and up to 37% in men [1]. In Germany, around ~58 kg bread is consumed per person annually, whereas bread consumption is significantly lower in countries with a typically Mediterranean diet [2].

However, review articles looking at undifferentiated bread consumption could not show a significant association between bread consumption and weight loss [3,3,4,5]. This might be due to the fact, that the variety of breads is huge, and bread is definitely not just bread. In addition to providing carbohydrates, bread is also an important source of fibre, proteins, minerals, vitamins and other bioactive compounds [6]. Bread baked from milled whole grain or refined wheat flour is characterised not only by high glycaemic but also high insulin indices [2], which are known to inhibit lipolysis [7]. High-carbohydrate diets and hyperinsulinaemia [8,9,10] are associated with being overweight and obese, type 2 diabetes and cardiovascular diseases [11,12,13,14]. Since a low-insulin-releasing lifestyle has been shown to lead to clinically relevant weight reduction (≥5%) in overweight or obese individuals [15] and to improve glucometabolic parameters in individuals with and without diabetes [16,17,18,19], reduction of digestible carbohydrates, which contributes to lower insulin levels [20,21], is a successful strategy for weight reduction [22]. In this context, prospective cohort studies have demonstrated that the long-term risk for being overweight or for obesity is associated with the consumption of less-complex and strongly processed bread types [23,24,25,26], whereas more complex and less processed bread is beneficial for reducing the risk of developing gastrointestinal and cardiovascular diseases, type 2 diabetes mellitus and certain types of cancer [23,24,27,28,29]. Therefore, conscious bread consumption might play an essential role in weight control.

So far, it is still unclear how exactly and to what extent bread consumption is related to regulation of body weight. In previous work [30] we could demonstrate that the carbohydrates in various types of bread cause heterogeneous levels of insulin secretion. However, intervention studies investigating the impact of low-insulin-stimulating bread on weight development and accompanying health parameters are lacking. Therefore, the aim of the present trial was to determine the insulin-stimulating potential of different bread types and concomitantly to prove the hypothesis that consumption of a low-insulin-stimulating bread compared to a conventional higher insulinogenic bread would lead to a significant difference in weight change in overweight adults.

## 2. Materials and Methods

### 2.1. Study Design and Participants

The triple-blind randomised controlled trial was conducted at the West German Centre of Diabetes and Health, Düsseldorf, Germany. Volunteers were recruited by newspaper report. Eligible participants were 18–69 years old, with body mass index (BMI) ≥ 27 kg/m^2^ and consumed bread on a daily basis; exclusion criteria were acute diseases, severe illness with in-patient treatment during the last 3 months, medication for weight reduction, weight change > 2 kg/week during the last month, smoking cessation during the last 3 months, or intolerance to components of the investigated breads. Between 1 August 2020, and 21 October 2021, 90 persons were screened, 6 were included in the pre-tests and 80 in the randomised controlled trial.

A three-stage procedure was followed to identify breads differing in the glucose and insulin response elicited: in the first pre-test volunteers meeting the entry criteria for the subsequent randomised controlled trial were equipped with a continuous glucose monitoring (CGM) system (FreeStyle Libre, Abbott Diabetes Care, Alameda, CA, USA). After an overnight fast participants consumed 50 g of different breads (*n* = 10) at the same time in the morning, in random order, on separate days. Foods and beverages throughout the rest of the day were not specified and self-chosen. Bread types were provided by a local bakery (Bäckerei Hinkel, Düsseldorf, Germany) or from STEINERfood GmbH, Sulz im Weinviertel, Austria.

### 2.2. Pre-Tests

Two breads with comparable texture but differing glucose-stimulating potential (i.e., the medium-carbohydrate, low-insulin-stimulating bread (intervention bread) and the rye bread from milled whole grain (control bread) were chosen for the second pre-test. As described before, 50 g study bread was consumed after an overnight fast on consecutive days. Glucose and insulin levels were determined in venous blood samples collected every 30 min over a period of 120 min after inserting an intravenous cannula into the forearm vein. Analyses were performed at the local laboratory [30].

### 2.3. Randomisation and Masking

For the randomised controlled trial, an unblinded statistician created the computer-generated randomisation list. Participants were equally allocated to the two groups. A closed and numbered envelope was handed out to the participants containing a verification card with coded information on the bread type for the local bakery. The study breads were baked with a comparable look and according to the verification card, the bakery employees handed out the control or intervention bread. The verification card was also used to note when and how many breads were picked up. Participants, investigators and the data analyst were blinded for group assignment. Participants were not aware of ingredients of the bread type received.

### 2.4. Procedures

Participants in the randomised controlled trial visited the study centre in fasting state, on the first day and after 3 months of intervention, for collection of anthropometric and clinical data (age, sex, body weight, height, BMI, waist circumference, blood pressure, as well as lean and fat mass). Body weight was measured in light clothing to the closest 0.1 kg, height to the closest 0.5 cm, and waist circumference at the minimum abdominal girth (about midway between the rib cage and the iliac crest). Body composition was measured using a state-of the-art body composition scale (Seca mBCA515, Seca, Hamburg, Germany). Blood pressure was determined on both arms in sitting position after a 5 min rest. Laboratory parameters were determined from venous blood samples at the local laboratory. Blood glucose was measured by photometry with an intra-assay coefficient of variability (CV) of 1.9%, and plasma insulin by electrochemoluminescence immunoassay (ECLIA) with an CV of 3.6%. Questionnaires were handed out at baseline and at follow-up to record physical activity and dietary habits during the study. Duration (0–1 h per week; 2–3 h per week; 4–5 h per week; >5 h per week) of physical activity (e.g., gardening or longer walks) was investigated via questionnaires. Dietary habits were split into three groups: vegetarian diet (i.e., fruits, vegetables, dried beans and peas, grains, seeds, and nuts, but also milk and eggs), mixed diet (i.e., potatoes, pasta, bread, meat, sausage, vegetables, salad, eggs, butter, cream), or Mediterranean diet (i.e., vegetables, fruit, salad, fish, less meat, pasta, bread, vegetable oils).

Participants picked up the breads from the local bakery, at weekly intervals, without learning about the nature of the bread type received. Participants were encouraged to eat as much bread as they normally would. No other breads, rolls or baked goods were allowed to be consumed during the 3-month intervention phase. The rye bread was made from type 997 flour, the low-insulin-stimulating bread consisted of oat flakes, sunflower seeds, flax seeds, chia seeds, psyllium husks, chopped almonds, baker’s honey, and Rhinish field beans.

### 2.5. Outcomes

For selecting the two bread types to be compared in the first pre-test, the incremental area under the curve (iAUC) of the postprandial blood glucose was calculated geometrically as the sum of the areas of the triangles and trapezoids over 120 min, excluding the area below the initial fasting concentration [30].

The primary outcome of the randomised controlled trial was the ETD in change in body weight between the two groups after 3 months. Secondary outcomes were the ETDs in change in BMI, hip circumference, waist circumference, blood pressure, triglycerides, total cholesterol, LDL cholesterol, HDL cholesterol, HbA1c, fasting blood glucose, fasting blood insulin, fat mass, and fat-free mass.

### 2.6. Statistical Analysis

The sample size calculation was based on the ‘double-sided two-sample analysis with continuity correction’ (SISA, Simple Interactive Statistical Analysis) method. Assumptions made for this calculation were based on previous nutrition studies [31], estimating a 1.0 ± 1.5 kg larger weight loss after 3 months in the intervention group who consumed the low-insulin-stimulating bread compared to the control group with the commonly consumed rye bread [22]. In order to identify such a weight reduction with a 1:1 randomisation, an accompanying power of 80%, a level of significance of 5%, and an estimated dropout rate of 10% [31], 40 persons per group had to be recruited.

Intention-to-treat (ITT) analyses were performed. Missing values (due to discontinued allocated intervention) were imputed by the ‘last observation carried forward’ (LOCF) principle. Non-normally distributed data were analysed by Mann–Whitney test for between-group comparisons and by Wilcoxon signed rank test for within-group comparisons. Differences in changes after 3 months between both groups were analysed using ANCOVA with adjustment for baseline values. Normality was visually and analytically confirmed by using histogram graphs and applying the Shapiro–Wilk test. Chi-square test was used to analyse dichotomous variables. All statistical tests were two sided, and the level of significance was set at α = 0.05. All analyses were performed using SPSS 22.0 (SPSS Inc., Chicago, IL, USA) and GraphPad Prism 6.04 (GraphPad Software, San Diego, CA, USA).

## 3. Results

The pre-test identified four breads with medium (14%) or low digestible carbohydrate (3–4%) content (Table 1), which differed significantly in post-load continuous monitoring glucose kinetics from six other breads with the usual digestible carbohydrate content of 38–54%.

**Figure 1:**
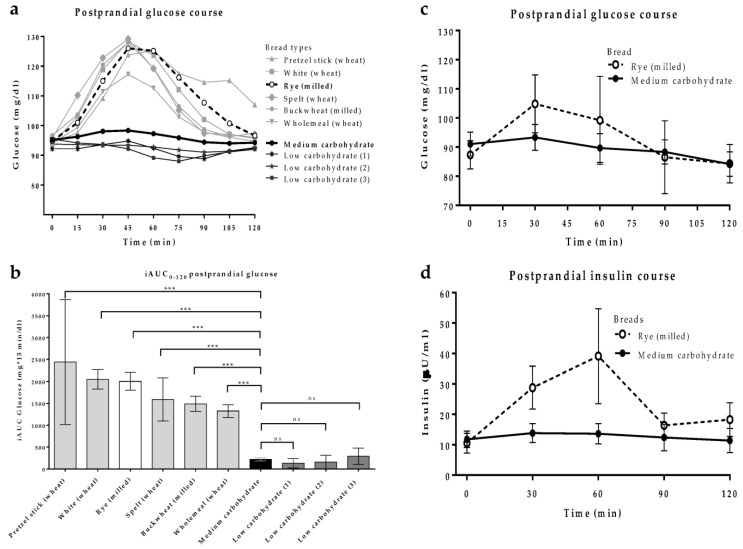
Postprandial glucose and insulin responses in the pre-tests. Participants (*n* = 6) consumed 50 g of ten different study breads on separate days after an overnight fast. (**a**) Mean glucose values measured by continuous glucose monitoring and (**b**) corresponding area under the curve for 0–120 min (mean ± SD). Mann–Whitney test was used for determining inter-group differences, *** *p* < 0 001. Participants (*n* = 6) consumed 50 g of the milled whole grain rye bread and of the medium-carbohydrate bread on consecutive days after an overnight fast and venous blood was collected every 30 min for 120 min. (**c**) Postprandial course of mean ± SEM blood glucose levels and (**d**) corresponding course of mean ± SEM blood insulin levels.

Whereas consumption of pretzel sticks, white, rye, spelt, buckwheat, and whole-meal bread provoked an increase in glucose levels of about 20–35 mg/dL, peaking after 45 min, ingestion of medium- and low-carbohydrate breads induced almost no significant glucose increase over 120 min (Figure 1a). In detail, glucose iAUC between 0 and 120 min was the highest after consumption of 50 g of pretzel stick, white, and rye bread (>2000 mg*15 min/dL each) and between 1300 and 1600 mg*15 min/dL for spelt, buckwheat, and whole-meal bread, respectively. In contrast, after consumption of medium- or low-carbohydrate breads, the iAUC just reached values of about 100–300 mg*15 min/dL (Figure 1b).

In order to examine the influence of bread consumption on blood glucose and insulin levels in more detail, for the second part of the pre-test (and the randomised controlled trial) the medium-carbohydrate, low-insulin-stimulating bread was chosen as intervention bread and the rye bread from milled whole grain as control. Measurement of glucose in venous blood confirmed the results observed during CGM. Venous glucose and insulin levels showed the expected rise after ingestion of the rye bread but there was no significant impact on glucose or insulin levels after uptake of the medium-carbohydrate bread with low-insulin-stimulating potential (Figure 1c,d).

**Figure 2:**
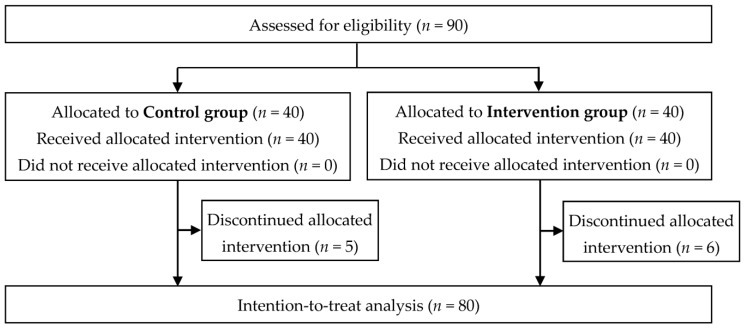
Flow chart.

Prior to the study, participants of both groups consumed a mean of 3.5 slices of bread per day, mostly wholemeal wheat and mixed rye–wheat (including buns). Baseline characteristics of participants participating in the randomised controlled trial were similar for both groups (Table 2) and 69 (86%) completed the allocated intervention (Figure 2).

**Figure 3:**
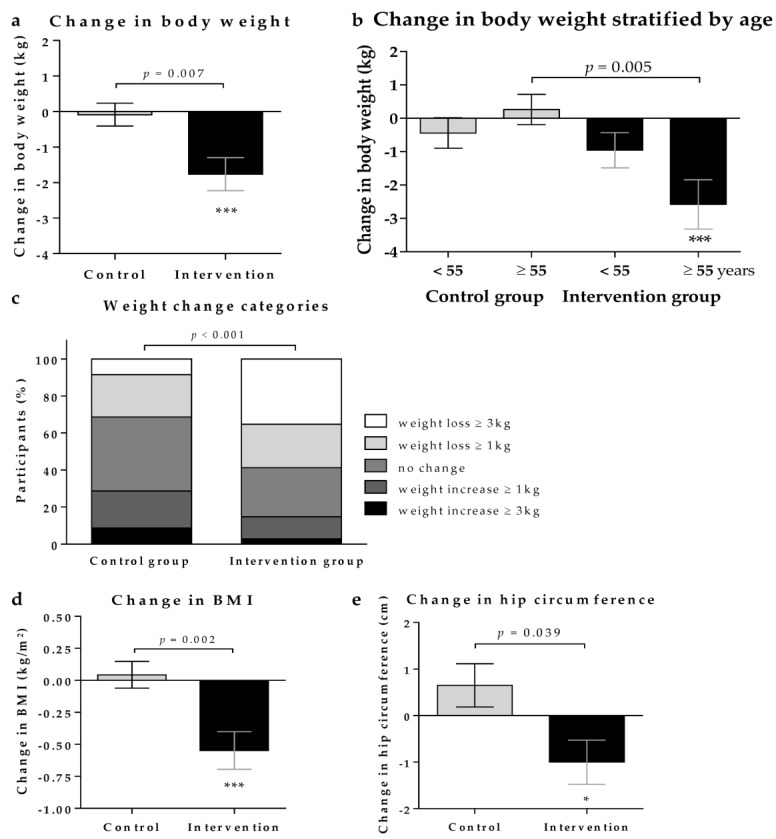
Effects on body weight, BMI and hip circumference. Changes in (**a**) body weight, (**b**) body weight stratified by the mean age of 55 years (**d**) BMI, and (**e**) hip circumference are shown as mean ± standard error of mean and compared using Mann–Whitney test for between-group differences and Wilcoxon signed rank test for within-group differences (* *p* < 0.05; *** *p* < 0.001); Chi-square test was used to analyse differences in (**c**) weight change categories between groups.

Whereas no change of body weight was observed in the control group (−0.1 ± 2.0 kg), significant weight reduction was observed in the intervention group (−1.8 ± 2.9 kg; *p* = 0.0003), with an ETD of −1.7 ± 0.2 kg (*p* = 0.007), who consumed the low-insulin-stimulating bread for 3 months (Figure 3). Stratification into two groups by the mean age of 55 years showed no significant weight change in the control group, neither in the participants below nor above 55 years. In the intervention group, a significant weight reduction (−2.6 ± 3.3 kg; *p* = 0.0007) was observed regarding the older participants, which significantly differed from the age-matched controls (*p* = 0.005). In the control group, a similar number of persons lost or gained weight, whereas, in the intervention group, about two thirds exhibited loss of body weight of at least 1 kg and about one third had lost ≥ 3 kg (*p* < 0.001 for the difference between groups). This was paralleled by a significantly stronger reduction in BMI and hip circumference in the intervention group, with ETDs of −0.5 ± 0.4 kg/m^2^ (*p* = 0.002) and −1.7 ± 1.4 cm (*p* = 0.039). No other statistically significant changes in clinical or biochemical parameters (Supplementary Material Table S1) or in lifestyle characteristics were observed.

Lifestyle characteristics with relevance for body weight development were evaluated at baseline and at the end of treatment in those who completed the trial. During the trial, the mean number of breads consumed per week was 1.4 [1.2; 1.6] for the control vs. 1.2 [1.0; 1.5] for the intervention group (*p* = 0.183). The shape of the study bread was the same, which relates to consumption of a similar number of 50 g slices consumed per day during the study (3.1 [2.6; 3.5] in the control vs. 3.4 [2.8; 4.1] in the intervention group; *p* = 0.262). Adverse events associated with eating either bread type were not reported. Physical activity in hours per week did not significantly change during the trial, nor was there a difference between the two groups. The type of diet consumed at baseline was generally maintained during the trial. The distribution of diet types consumed did not differ between control and intervention groups.

## 4. Discussion

Since data about the effects of bread on weight development are inconsistent, we analyzed the effect of different bread types on glucose and insulin levels and compared the effects of consumption of a medium-carbohydrate, low-insulin-stimulating bread vs. a conventional rye bread from milled grain, as control, on the weight change in overweight persons in a 3-stage randomised controlled trial. CGM, as used as a scientific approach for nutritional analyses, demonstrated that the postprandial glucose courses after consumption of medium- or low-carbohydrate breads were significantly lower compared to conventional bread types. Analyses of insulin levels showed that postprandial deflections were also diminished after consumption of the intervention bread vs. control bread. Without further lifestyle changes, consumption of low-insulin-stimulating bread for 3 months led to a significant weight reduction in the intervention group—more pronounced in participants ≥ 55 years—compared to stable weight in the control group, resulting in an ETD of −1.7 ± 0.2 kg (*p* = 0.007). Thus, consumption of low-insulin-stimulating bread might be an effective and low-threshold entry into a lifestyle intervention for overweight people, especially those at older age.

Bread is a typical component of the average diet in Germany [1,2] which is reflected by a mean of 3.5 slices of bread consumed daily by the participants prior to the trial. This translates to about 100–200 g of bread per day, which perfectly fits to the estimated consumption of about 58 kg bread per person a year in Germany [32]. In countries with a typically Mediterranean diet, bread consumption is significantly lower, around 46 kg in Spain and 44 kg in Italy. In the United States mean bread consumption is also around 43 kg [32] and it provides less than 15% of daily carbohydrate intake [33,34], whereas in Germany it accounts for 13–30% of daily carbohydrate intake in women and 14–37% among men [1]. As a result of conscious bread consumption, daily carbohydrate intake could be reduced in Germany; whereas, in other countries with less bread consumption, the effects might be lower.

The low-insulin-stimulating bread contained less starch than the milled whole grain rye bread. Its lower energy content was made up for by a higher fat content. Since fibre, which is present in whole grain but not in refined wheat flour, has beneficial health effects [35] the fibre content of the bread types analysed was kept similar and, moreover, there were no major differences regarding protein content, texture and taste between the two study breads. We therefore assume that the different metabolic response to the low-insulin-stimulating bread was of relevance. We had selected this bread type for comparison with a usual bread based on the absence of a detectable rise in blood glucose and insulin levels after consuming 50 g bread. Lowering the insulin response to meals has been reported previously to lower body weight in randomised controlled trials [15,16,36]. Pharmacological lowering of circulating insulin levels by diazoxide or octreotide also led to body weight reduction in most trials [9]. In mice, genetic lowering of the number of insulin genes expressed and of circulating insulin levels prevented or partially reversed diet-induced obesity [37]. The relevance of insulin levels for body weight regulation has led to the carbohydrate-insulin-concept [22,38] and mirrors results of meta-analyses that found low-fat diets inferior to low-glycaemic diets for weight reduction [39,40]. As shown in our study, when eating the three low-carb bread types, an immensely reduced glucose rise can be achieved by replacing flour from grain with protein, non-cereal flour and fibre. Studies using a wide variety of functional additives confirm the success of this approach [32,41,42] while, at the same time, a down-regulation of the appetite was observed [32].

Although the association between elevated insulin levels and obesity initiated the concept of the current study protocol, the trial did not aim to test a hypothetic metabolic mechanism or to determine the glycaemic index (GI) or the glycaemic last (GL) of the study breads. Rather, it tested whether simply exchanging one common dietary component with insulin stimulatory properties for a poorly insulin-inducing alternative would have an impact on body weight development in the absence of any recommendation to alter the daily diet composition or other aspects of participants’ lifestyles. Therefore, we can only speculate that the differences in weight development found emphasize the importance of low-insulin nutrition, especially in the elderly. Studies have shown that, with increasing age, the fasting insulin levels (measured by C-peptide) and the glucose-induced insulin secretion increase [43] and usually weight does too.

There are some strengths and limitations that need to be mentioned. The concept of the trial was to substitute the usual bread type, as an insulinogenic component of the diet, with a similar, but low-insulin-stimulating, alternative in a setting that mimicked real-world conditions. The strength of this setting is that the participants adhered to their usual lifestyle, i.e., daily eating habits and physical activity level. To keep awareness of the trial situation as low as possible, they had no contact with the study centre during the intervention period, and there was no request for repeated documentation of diet or lifestyle characteristics during the trial. Analysis of responses to questionnaires before and after the intervention phase indicated the absence of a recognisable study effect regarding the amount of bread consumed, the overall diet, and physical activity.

On the other hand, the real-world approach prevents the control of certain influences. As with every lifestyle study, there is a certain uncertainty in adherence measurement. In order to estimate the adherence, we counted the amount of bread that the participants picked up in the bakery and also asked the participants, using self-disclosure questionnaires, how many slices of bread they ate per day from the study bread; these values matched well. If an absolute degree of adherence measurement is needed, real-world studies would not be possible, and the effects could only be examined under laboratory conditions. However, the question is whether the results could be transferred in real life. Owing to the study design, the insulin-stimulating potential of the pre-study consumed breads was not measured. However, to get this information a study with an immense number of participants would have been needed. Nevertheless, concrete statements about individual breads would only have been possible to a limited extent since, although the study participants mostly consumed the same type of bread, they sometimes also ate a roll or other baked goods. Since the study was carried out during the Corona pandemic, which generally was associated with reduced physical activity and weight gain, this is not reflected in our data. In the intervention group, the proportion of people with a high level of physical activity tended to decrease and the proportion of inactivity tended to increase, but in the control group this was rather the opposite. Because of the randomisation into parallel groups, seasonal effects should have no impact on the differences between groups. However, these differences were neither statistically significant between the groups, nor in the course of the trial, and would have rather led to an underestimation of the effect.

We decided to compare the effect of 50 g of different types of bread in our randomised controlled trial. Since the breads consist of different ingredients, it could be said that the carbohydrate content was different, which could also be reflected in the glucose and insulin profiles. However, there are only two ways of doing such an analysis, i.e., either equating the carbohydrate content and then serving different portion sizes or, conversely, standardizing the portion sizes in the knowledge of different carbohydrate content. We chose the latter because simultaneous questioning demonstrated that eating bread is a kind of “ritualized” process and participants ate an equal amount of bread each day. It is also, therefore, more realistic to advise persons to eat, e.g., two slices of whole-grain bread instead of eating whole-grain bread such that you consume 50 g of carbohydrates. This is also the problem with the common measurement methods for carbohydrate quality, since for calculating the GI, an amount of a food must be consumed that contains 50 g of digestible carbohydrates [44]. In addition, GI is influenced by numerous factors, such as food composition, processing, and preparation, so that its application is inherent in practice.

By measuring glucose in venous blood, and via CGM in interstitial fluid, reliable statements about the postprandial glucose increase after bread meals can be made. However, there were methodological differences between the pre-tests: using CGM glucose levels were measured every 15 min, whereas venous blood was only taken every 30 min. In this way, it seems likely that we missed the maximum peak (after 45 min) in glucose and insulin after consumption of rye–wheat bread. In the case of the low-insulin-stimulating bread, this is obviously not the case since there was no appreciable increase in glucose and insulin levels. Overall, this led to an underestimation of the observed differences.

## 5. Conclusions

Simply exchanging a common insulinogenic bread for a low-insulin-stimulating bread demonstrates potential to induce weight loss in overweight persons. To the best of our knowledge, this is the first triple-blind randomised controlled trial comparing two different bread types, with contrasting insulin-releasing effects, for an influence on weight development over 3 months, in a study population used to daily consumption of bread. Whether the effects of exchanging the bread type for body weight reduction additionally impact insulin sensitivity or can be extended to other common dietary components with insulin-stimulating properties requires further study. In summary, consumption of low-insulin-stimulating bread can be an effective and low-threshold entry into a lifestyle intervention for overweight people, especially those of older age.

## Figures and Tables

**Table 1:** Composition of bread types.

Breads (100 g)	Pretzel Stick ^1^	White ^1^	Rye ^2^	Spelt ^1^	Buck Wheat ^2^	Whole- Meal ^1^	MC	LC (1)	LC (2)	LC (3)
Energy (kcal)	264	272	217	233	237	251	237	212	206	199
Energy (kJ)	1120	1153	917	985	998	1062	989	887	862	830
Carbohydrate (g)	49	54	44	42	49	38	14	3.0	4.4	3.0
Sugar (g)	1.2	1.4	0.8	0.8	1.2	7.0	1.9	1.7	0.9	1.6
Total fat (g)	3.7	1.7	0.8	1.7	4.8	1.2	14.6	7.8	7.8	5.9
Saturated fatty acids (g)	2.0	0.7	0.2	0.2	0.6	<0.2	0.8	1.2	2.8	1.0
Total protein (g)	7.8	8.4	5.4	9.2	8.3	6.7	8.8	28	26	28
Fiber (g)	4.0	3.0	6.0	4.0	3.0	8.0	7.8	14	9.7	12.5
Sodium (g)	3.1	1.8	1.6	1.2	1.6	2.0	0.4	1.5	1.3	1.6

^1^, wheat; ^2^, milled; MC, medium-carbohydrate; LC, low-carbohydrate.

**Table 2:** Baseline characteristics.

	Control Group (*n* = 40)	Intervention Group (*n* = 40)
Sex (%) ♀/♂	62/38	45/55
Age (years)	55 ± 11	55 ± 8
Weight (kg)	98 ± 15	102 ± 24
BMI (kg/m^2^)	33.1 ± 4.2	34.1 ± 6.9
Hip circumference (cm) ♀/♂	120 ± 12/115 ± 7	120 ± 15/115 ± 11
Waist circumference (cm) ♀/♂	107 ± 11/117 ± 8	107 ± 12/119 ± 15
Fat mass (%)	42 ± 7	40 ± 8
Fat-free mass (%)	58 ± 7	60 ± 8
Systolic blood pressure (mmHg)	120 ± 10	123 ± 12
Diastolic blood pressure (mmHg)	68 ± 6	68 ± 7
Fasting blood glucose (mg/dl)	94 ± 12	103 ± 24
Fasting insulin (µU/mL)	12.1 (8.3; 18.2)	16.1 (8.7; 23.1)
HbA1c (%)	5.6 ± 0.4	5.8 ± 0.6
Cholesterol (mg/dl)	214 ± 36	207 ± 41
HDL cholesterol (mg/dl)	57 ± 15	53 ± 13
LDL cholesterol (mg/dl)	139 ± 35	137 ± 35
Triglycerides (mg/dl)	129 (80; 173)	134 (97; 198)
Antidiabetic drugs (%)	3	5
Antihypertensive drugs (%)	30	33
Statins (%)	8	3
Smoker (none/former/active) (%)	82/15/3	73/21/6
Physical activity (0–1/2–3/4–5/>5 h/week) (%)	20/42.5/20/17.5	12.5/30/32.5/25
Diet (vegetarian/western/mediterranean) (%)	3/85/12	6/87/7
Bread (slices per day)	3.5	3.5

Data are presented as mean ± standard deviation, median (interquartile range: 25th; 75th) or percentages. ♀, female; ♂, male; BMI, body mass index; HDL, high-density lipoprotein; LDL, low-density lipoprotein.

## Data Availability

Data are available from the corresponding author on request.

## Supplementary Materials

The following supporting information can be downloaded at: https://www.mdpi.com/article/10.3390/nu15051301/s1, Table S1: Differences after 3 months.
