# Supplementary material for: Impact of a Low-Insulin-Stimulating Bread on Weight Development—A Real Life Randomised Controlled Trial
Source: Nutrients. 2023 Mar 6;15(5):1301. doi: 10.3390/nu15051301 (PMC10004839; doi:10.3390/nu15051301)
Supplement: Supplementary file 1 [file nutrients-15-01301-s001.zip › nutrients-2271424-supplementary.pdf]

Supplementary Material

Table S1: Differences after 3 months.

	**Control group (n=40)**	**Intervention group (n=40)**
Waist circumference (cm)	-0.5 [-1.7; 0.8]	-0.3 [-1.4; 0.8]
Fat mass (kg)	-0.4 [-1.1; 0.4]	-0.6 [-1.4; 0.2]
Fat free mass (kg)	-0.3 [-1.0; 0.3]	-1.0 [-1.7; 0.1]
Systolic blood pressure (mmHg)	0.3 [-2.8; 3.4]	0.3 [-2.9; 3.4]
Diastolic blood pressure (mmHg)	-1.5 [-3.2; 0.2]	0.6 [-1.1; 2.4]
Fasting blood glucose (mg/dl)	0.3 [-2.8; 3.2]	1.3 [-1.9; 4.4]
Fasting insulin (µU/ml)	0.4 [-2.0; 2.8]	1.5 [-0.9; 3.9]
HbA1c (%)	-0.04 [-0.09; 0.02]	-0.05 [-0.10; 0.00]
Cholesterol (mg/dl)	1 [-6; 7]	1 [-5; 8]
LDL cholesterol (mg/dl)	1 [-1; 3]	0 [-3; 2]
LDL cholesterol (mg/dl)	-4 [-10; 1]	-3 [-9; 3]
Triglycerides (mg/dl)	4 [-13; 20]	5 [-12; 21]
Physical activity (0-1/2-3/4-5/>5 h/week) (%)	12.5/30/12.5/45	22.5/27.5/27.5/12.5

Data are shown as mean [95% CI]. Differences in changes after 3 months between both groups were analysed using ANCOVA adjusting for baseline values.
